# Potential Role of the Gut/Liver/Lung Axis in Alcohol-Induced Tissue Pathology

**DOI:** 10.3390/biom5042477

**Published:** 2015-09-30

**Authors:** Veronica L. Massey, Juliane I. Beier, Jeffrey D. Ritzenthaler, Jesse Roman, Gavin E. Arteel

**Affiliations:** 1 Departments of Pharmacology and Toxicology and the University of Louisville Alcohol Research Center, University of Louisville Health Sciences Center, Louisville, KY 40292, USA; E-Mails: veronica_massey@med.unc.edu (V.L.M.); juliane.beier@louisville.edu (J.I.B.); j0roma02@exchange.louisville.edu (J.R.); 2 Department of Medicine University of Louisville Health Sciences Center, Louisville, KY 40292, USA; E-Mail: j.ritzenthaler@louisville.edu

**Keywords:** ethanol, hepatic, pulmonary, inflammation, extracellular matrix

## Abstract

Both Alcoholic Liver Disease (ALD) and alcohol-related susceptibility to acute lung injury are estimated to account for the highest morbidity and mortality related to chronic alcohol abuse and, thus, represent a focus of intense investigation. In general, alcohol-induced derangements to both organs are considered to be independent and are often evaluated separately. However, the liver and lung share many general responses to damage, and specific responses to alcohol exposure. For example, both organs possess resident macrophages that play key roles in mediating the immune/inflammatory response. Additionally, alcohol-induced damage to both organs appears to involve oxidative stress that favors tissue injury. Another mechanism that appears to be shared between the organs is that inflammatory injury to both organs is enhanced by alcohol exposure. Lastly, altered extracellular matrix (ECM) deposition appears to be a key step in disease progression in both organs. Indeed, recent studies suggest that early subtle changes in the ECM may predispose the target organ to an inflammatory insult. The purpose of this chapter is to review the parallel mechanisms of liver and lung injury in response to alcohol consumption. This chapter will also explore the potential that these mechanisms are interdependent, as part of a gut-liver-lung axis.

## 1. Introduction

### 1.1. The Global Impact of Alcohol Consumption on Human Health

Alcohol consumption is customary in most cultures and alcohol abuse is common worldwide. For example, more than 50% of Americans consume alcohol, with an estimated 23.1% of Americans participating in heavy and/or binge drinking at least once a month [1]. The detrimental effects of drinking alcohol, particularly heavy and/or chronic consumption, are well-established. The most commonly recognized symptoms of alcohol consumption are associated with chronic alcoholism, and it is a causal/risk factor in over 60 major types of diseases. These and other effects of alcohol consumption have made alcohol the third leading risk factor, globally, for disease and disability [2]. The cost of medical consequences of alcohol abuse has been projected to be more than 20 billion dollars annually in the US, making it a significant financial burden for the health care system [3]. These estimates take into account only direct medical expenses; it is estimated that the cost of lost productivity due to alcohol-related illness is >150 billion dollars annually in the US. Thus, the medical consequences of alcohol consumption have significant societal consequences in addition to the effects on the individual.

### 1.2. Alcohol and Liver Diseases

The liver is the main site of alcohol metabolism and a major target organ of alcohol-induced injury. The susceptibility of the liver to alcohol-induced toxicity is due to both the high concentrations of alcohol found in the portal blood (*versus* systemic), as well as the metabolic consequences of ethanol metabolism. The risk of alcoholic liver disease (ALD) increases in a dose- and time-dependent manner with consumption of alcohol. ALD ranks among the major causes of morbidity and mortality in the world [4], and affects millions of patients worldwide each year. It is estimated that one in three liver transplants performed in the United States is due to ALD [5].

Progression of ALD is well-characterized and is actually a spectrum of liver diseases, which ranges initially from simple steatosis, to inflammation and necrosis (steatohepatitis), to fibrosis and cirrhosis. One effective therapy for ALD is orthotopic liver transplantation (OLT) [6]. However, the usefulness of OLT as a general therapy is limited, owing to a well-documented donor organ shortage, as well as ethical issues concerning the treatment of inveterate alcoholics. A major focus in ALD therapy is to treat the decompensation associated with the disease. Indeed, the sequelae of a failing liver (e.g., ascites, portal hypertension, and hepatorenal syndrome) are generally what cause death in end-stage liver disease [6]. Although the successful treatment of these secondary effects prolongs the life of ALD patients, this therapy is only palliative. Furthermore, since underlying cirrhosis greatly increases the risk of developing hepatocellular carcinoma (HCC) [7], success in maintaining “stable cirrhotics” may translate into an increase in the incidence of HCC. Indeed, HCC incidence is increasing in the US and in Europe [8]. Once a person develops HCC, the survival rate is almost nil [9].

### 1.3. Alcohol and Lung Disease

Alcohol abuse is known to increase the risk for lung disease. However, the impact of acute and chronic alcohol exposure to the pulmonary system is poorly understood. Alcohol has been shown to increase the risk for lung infection with tissue-damaging Gram-negative pathogens such as *Klebsiella pneumonia* [10] or for the spread of bacteria in the blood (*i.e.*, bacteremia) and shock from pathogens, most notably *Streptococcus pneumonia* [11] and *Mycobacterium. tuberculosis* [12]. Similar observations have been made in animals. In rats, for example, alcohol feeding is associated with increased spread of *S. pneumonia* and *K. pneumonia* from the lung via the bloodstream and failure to clear the infection [13,14,15]. Thus, chronic alcoholics are thought of as “immunocompromised hosts” because of increased incidence and severity of infections.

Many factors contribute to the susceptibility to infection in alcoholics including their increased risk for aspiration of gastric contents and/or microbes from the upper respiratory track (*i.e.*, oropharyngeal flora) as well as decreased mucous-facilitated clearance of bacterial pathogens from the upper airway [16]. Furthermore, the prevalence of oropharyngeal colonization with *K. pneumoniae* may be four times higher in alcoholics compared with non-alcoholics [17]. In addition, the normal gag and cough reflexes, along with other upper airway clearance mechanisms, have been shown to be depressed in alcoholics, thereby enhancing the risk of colonization and aspiration. In experimental animal models, alcohol ingestion disrupts the normal beating motion of the cilia that is important in the clearance mechanisms aimed to help remove pathogens from the airways [18]. These abnormalities, together with documented impairments in pulmonary host defenses due to defects in neutrophil and macrophage functions [19], explain the increased infection rate observed in alcoholics. However, the effects of acute *versus* chronic alcohol exposure remain unclear and available data suggest that there are distinct effects. For example, a bolus of injection of alcohol in animals results in increased neutrophil chemotaxis to fMLP [20], while there is decreased chemotaxis in neutrophils harvested from chronic alcoholics [21,22].

Although the link between alcohol exposure and lung infection has been known for decades, it was not until 1996 when the broader impact of alcohol on the lung began to be unraveled after Moss *et al.* reported that alcoholics are more susceptible to the development of the acute respiratory distress syndrome (ARDS) when exposed to at-risk factors such as sepsis [23]. ARDS is the most severe form of acute lung injury with an incidence close to 200,000 cases per year in the U.S. and with a mortality rate of around 40% [24]. Moss *et al.* [23,25] demonstrated that ARDS occurs 3.7 times more often in people who meet the diagnostic criteria for alcohol use disorders. These studies, as well as others performed subsequently, raised awareness about the link between chronic alcohol abuse and acute lung injury, and launched a new era of investigation directed at exploring the mechanisms by which alcohol affects lung inflammation and tissue remodeling.

Although *in vitro* studies have been very helpful in elucidating mechanisms responsible for the effects of alcohol in lung, the most informative work has been performed in animal models of alcohol exposure. These studies, modeled after well-described animal models of alcohol-related liver disease, have unveiled abnormalities in lung structure and function that explain, at least in part, the increased incidence of acute lung injury in alcoholics. For example, in rodents, chronic exposure to alcohol (up to 6–8 weeks) is associated with up-regulation of pro-inflammatory cytokines [26], disruption of normal regulatory signaling pathways [27], activation of tissue remodeling [28,29], and the induction of oxidative stress [30]; all of which contribute to the production of the “alcoholic lung phenotype”. It is this phenotype that appears to render the host highly susceptible to pulmonary injury caused by respiratory infections along with other serious lung diseases like acute lung injury [31,32].

### 1.4. Alcohol Abstinence as a Therapy

Abstinence from alcohol is vital in order to prevent further ongoing organ damage. Abstinence from alcohol is beneficial for patients in all stages of ALD and is necessary to prevent progression of liver injury in those with early stages of disease [6]. Abstinence even improves outcomes in end-stage liver disease. Indeed, abstinence even dramatically improves 10 year survival in decompensated cirrhotics [33]. Although changes to the lung caused by chronic alcohol abuse (e.g., altered GSH pools, see Section 3.3) do not recover from short (one week) abstinence [34], it is likely that prolonged abstinence would also be beneficial in preventing ARDS. Unfortunately, a high rate of recidivism among alcohol abusers greatly reduces the opportunity for disease remission/therapy via abstinence. Therefore, better understanding of the mechanisms by which alcohol damages the liver may yield new pharmacologic strategies to blunt, halt, or reverse disease progression, potentially even in inveterate alcoholics. Gaps in our knowledge of how the diseases progress (and regress) must be filled before rational targeted therapies can be developed.

### 1.5. Purpose of This Review

In general, derangements seen in ALD and alcohol-related lung injury are considered to be independent of each other and, consequently, the effects of alcohol on these two organs (liver and lung) are often evaluated separately. However, the liver and lung share many general responses to damage. Both organs possess resident macrophages that play key roles in mediating the immune/inflammatory response. Also, alcohol-induced damage to both organs appears to involve oxidative stress [32,35]. Another mechanism that appears to be shared between the organs is that inflammatory injury to both organs is enhanced by alcohol exposure [29,30,36,37]. Lastly, altered extracellular matrix (ECM) deposition appears to be a key step in disease progression in both organs. In this review, we will explore not only the parallelism of these mechanisms of alcohol-induced liver and lung injury, but also their potential interdependence. A particular focus will be placed on the potential role of the gut:liver:lung axis in alcohol-induced liver and lung injury.

## 2. Alcoholic Liver Disease-Mechanisms of Injury

Although there is no universally-accepted mechanism-based therapy available to halt or reverse ALD, major advances have been made in our understanding of the mechanisms for the development and progression of ALD. The initiation and progression of ALD involves multiple resident and recruited cell-types that mediate a synergistic impact on hepatic health and function. Hypothesized general mechanisms involved in the development and progression of ALD include: (1) alcohol and alcohol metabolism; (2) dysregulated inflammation; (3) oxidative stress; and (4) altered extracellular matrix (ECM) metabolism. Each of these general mechanisms will be discussed in detail.

### 2.1. Alcohol and Hepatic Alcohol Metabolism

As mentioned above, alcohol exerts a tremendous metabolic demand on the liver. The liver is by far the main organ responsible for metabolizing alcohol. The major route of metabolism is the oxidation of alcohol to acetaldehyde. Concentrations of alcohol can easily reach the mM range in the portal/hepatic circulation during alcohol consumption. Whereas the dominant enzyme system involved in this process is alcohol dehydrogenases (ADH), the cytochrome P450 systems, also called microsomal alcohol oxidizing system or “MEOS” (mostly CYP2E1), and catalase also play critical roles. Whereas the oxidation of alcohol to acetaldehyde is mediated by three distinct enzyme systems, aldehyde dehydrogenase (ALDH) is essentially the only enzyme system that oxidizes acetaldehyde to acetate. Analogous to ADH, ALDH uses NAD^+^ as the electron acceptor for this reaction; however, ALDH is located in the mitochondrion of the cell. In the process of metabolizing alcohol to acetate, two equivalents of reduced NADH are generated per equivalent of alcohol oxidized. This metabolism robustly increases the ratio of NADH:NAD^+^ within the cell, when then inhibits the β-oxidation of fatty acids in the liver. Furthermore, ethanol metabolism also increases the rate of esterification of fatty acids [38]. The net effect is to favor fat accumulation in the hepatocytes.

Whereas acetaldehyde is subsequently oxidized to acetate by ALDHs, the kinetics of this reaction are sufficiently slow to allow for accumulation of acetaldehyde in humans consuming alcohol. Acetaldehyde is toxic and a number of the systemic toxic effects of alcohol abuse (e.g., flushing, headaches, and nausea) are mediated, at least in part, by direct or indirect effects of elevated acetaldehyde levels. At the more local level, it is proposed that acetaldehyde may also play an etiologic role in ALD [39]. For example, acetaldehyde can form adducts with reactive residues on proteins or small molecules (e.g., cysteines). These chemical modifications can alter and/or interfere with normal biologic processes and be directly toxic to the cell. Antibodies against such oxidatively-modified proteins have been reported in both humans and animal models of ALD [40,41,42]. Single nucleotide polymorphisms (SNPs) in the ADH genes have been identified that result in functional differences in the kinetic properties of the enzymes and the relative rate of alcohol metabolism [43]. Indeed, correlations between lower ALDH activity and increased risk for ALD have been made (see [44] for review).

Although the relative contribution of the MEOS system to total alcohol metabolism is low *a priori*, CYP2E1 is robustly induced by alcohol and can contribute to a far greater amount of total alcohol metabolism in alcohol-dependent individuals [45]. This induction may be viewed as a protective effect at the organismal level, it may however have side effects that contribute to hepatotoxicity. For example, selective inhibitors of CYP2E1 partially blocked hepatic pathology caused by alcohol in animal models, supporting this hypothesis [46]. The potential mechanisms by which CYP2E1 contributes to ALD are still being explored; however, there are some viable hypotheses. For example, CYP2E1 has been shown to be relatively loosely coupled with cytochrome reductase; it can, therefore, leak electrons to oxygen to form O_2_^−^, or catalyze lipid peroxidation [47]. It is therefore proposed that this enzyme may contribute to oxidative stress caused by alcohol (see below). Furthermore, CYP2E1 also can bioactivate a number of hepatotoxic agents (e.g., acetaminophen); the induction of this enzyme by chronic abuse of alcohol can therefore increase the risk of liver damage by other agents.

### 2.2. Dysregulated Hepatic Inflammation

Alcohol exposure impacts the inflammatory/immune response via a myriad of mechanisms. Alcohol preexposure can either tolerize or sensitize the liver to a second “hit” of bacterial lipopolysaccharide (LPS) [48,49]. Specifically, if LPS is administered during acute alcohol intoxication, it decreases hepatic inflammation and injury caused by LPS; in contrast, if LPS is given approximately one day after alcohol exposure or after chronic alcohol exposure, the inflammatory response is synergized. For example, alcohol intoxication (*i.e.*, when alcohol is present in the system) impairs the immune response. A major mechanism by which alcohol intoxication can affect inflammatory/immune responses is via altering Toll-like receptor (TLR) signaling, which serve as pattern recognition receptors on macrophages and other cells of the innate immune system [50]. TLRs are involved in both direct cytotoxic and effector responses of macrophages and, therefore, serve as key early initiators of an appropriate immune response. Importantly, alcohol intoxication has been shown to blunt the stimulation of macrophages by a number of TLR ligands, including zymosan A (TLR2) and LPS (TLR4) [51,52].

Although alcohol blunts the immune response during the intoxication phase, the effects of alcohol on the inflammatory response is to prime the cells to release cytotoxic mediators. The natural history of ALD is characterized by chronic, low-grade, inflammation in the liver. This activation of the inflammatory response can be at least partially attributed to increased levels of activators of this response, such as LPS [53,54]. However, the levels of LPS found in alcoholics and in experimental ALD are relatively low compared to those found in endotoxemia; furthermore, damage to liver caused by alcohol cannot be mimicked by chronic low-dose LPS in the absence of alcohol [55]. Rather, inflammatory cells appear to be primed to activation by alcohol administration. For example, peripheral blood monocytes obtained from patients with alcoholic hepatitis spontaneously produce pro-inflammatory mediators (e.g., TNFα), and they produce more of these mediators in response to LPS than do control monocytes [56]. Other cytokines and chemokines that have been shown to be elevated in ALD patients include IL-6 [57], IL-8 [58], MCP-1 [59] and MIP-1 [60]. In addition to cytokine/chemokine production, there are a host of other mediators of inflammation that are increased in ALD, such as adhesion molecule expression, ROS/RNS, and cytokine receptors (e.g., TNFR1).

In addition to priming inflammatory cells, liver cells appear to be sensitized to inflammatory stimuli by alcohol administration. For example, although TNFα is normally pro-proliferative in hepatocytes isolated from naïve animals, it is pro-apoptotic in cells isolated from alcohol-treated animals [61]. This effect appears to be mediated through the cellular “death domain” pathways [62]. The concept of priming and sensitization also implies that there may be a series of sequential events in the progression of liver injury during alcohol exposure. Specifically, the priming of inflammatory cells by alcohol leads to a more robust cell-killing response that is exacerbated in sensitized hepatocytes. The hepatocyte death further stimulates the inflammatory response, yielding a “vicious cycle”.

### 2.3. Oxidative Stress

Reactive oxygen and nitrogen species (ROS and RNS, respectively) are products of normal cellular metabolism and have beneficial effects (e.g., cytotoxicity against invading bacteria). However, due to the potential of these molecules also to damage normal tissue, the balance between pro-oxidants and antioxidants is critical for the survival and function of aerobic organisms. If the balance is tipped to favor overproduction of these species, oxidative stress can occur. Oxidative stress has been proposed to be critically involved in ALD [35,63,64]. Oxidative stress associated with clinical ALD is most likely not solely due to increased pro-oxidant formation. Alcoholics replace up to 50% of their total daily calories with alcohol, [65] leading to nutritional deficiencies that can be further complicated by nutrient malabsorption [66]. The net effect is that alcoholics often have lower levels of key dietary antioxidant molecules [67], and an overall decreased antioxidant status. Therefore, oxidative stress in ALD is most likely mediated both by an increase in pro-oxidant production, as well as by a decrease in antioxidant defenses. In support of the hypothesis that oxidative stress is involved in ALD, numerous antioxidants have been shown to protect against the damaging effects of alcohol *in vitro* and *in vivo* models of ALD [68,69,70].

Clearly, the most obvious pathologic changes to liver during alcohol exposure occur in the hepatocytes. Moreover, the accumulation of indices of oxidative stress (e.g., lipid peroxides) is predominantly a hepatocellular event during alcohol administration. Thus, it is likely that oxidant production by this cell type plays a critical role in alcoholic liver injury. Whereas there are also many potential sources of pro-oxidants, two major suspected sites in hepatocytes are the alcohol-inducible CYP2E1 (see Section 2.1, above) and mitochondria. It is now clear that the reduction of O_2_ to H_2_O by the mitochondria is not complete and that 1%–2% of O_2_ consumption by mitochondria leads to the formation of O_2_**^−^** [71]. Alcohol exposure increases the yield of O_2_**^−^** from this cellular component in the liver [72]. Moreover, alcohol depletes of mitochondrial GSH levels [73], which increases the response of hepatocytes to apoptotic stimuli [61]. Therefore, it is likely that pro-oxidant production from this cellular compartment is key for the development of severe ALD.

A key component in the progression of alcohol-induced liver injury is inflammation, involving both resident (e.g., Kupffer cells) and recruited (e.g., neutrophils and lymphocytes) inflammatory cells. In contrast to parenchymal cells, where ROS production is predominantly caused by electron leakage from biochemical processes, inflammatory cells are “professional” producers of ROS/RNS, capable of generating high concentrations of both types of species into their surrounding environment. Whereas the production of these species is critical for host defense, if inappropriately stimulated, they can also cause damage to normal tissue (see Section 2.2). Two major sources of pro-oxidants in these cells include NAD(P)H oxidase and the inducible form of NOS (iNOS or NOS2). Other sources of ROS in macrophages include xanthine oxidase [70], and myeloperoxidase [74]. The induction of inflammation and oxidative stress can initiate a vicious cycle that leads to progressive tissue damage.

### 2.4. Altered Hepatic Extracellular Matrix Metabolism

When an organ is chronically damaged from multiple hits, this injury often overwhelms the ability of the organ to recover and rebuild from the damage. Under such conditions, the organ often remodels in response to the damage. Even in the case of liver, which is well-known for its ability to regenerate, remodeling (*i.e.*, fibrosis) is a common response to chronic inflammatory liver injury. Remodeling of the liver is well-known during the fibrotic phase of ALD and is characterized by extracellular matrix (ECM) deposition. Although the main ECM characterized during fibrosis is collagen type I, other ECM proteins also accumulate, including laminin, fibronectin, and fibrin(ogen) [75]. The major cell type that contributes to fibrogenesis is the hepatic stellate cell that has transdifferentiated into a myofibroblast-like phenotype. However, other cellular origins of myofibroblast-like cells are becoming increasingly recognized, such as periportal fibroblasts, fibrocytes, and transdifferentiated epithelia [76,77,78,79]. Recent studies have demonstrated that in humans, hepatitis C virus (HCV)-induced liver fibrosis can be reversed if the underlying infection is effectively treated [80]. This exciting finding raises the prospect that advanced liver disease due to alcohol may also be reversible if the appropriate therapy can be identified. Nevertheless, hepatic fibrosis caused by ALD is viewed as the beginning of “end-stage liver disease” at this time.

More recent studies indicate that hepatic remodeling and ECM accumulation may also contribute to earlier phases of ALD, such as steatohepatitis. For example, work by this group and others recently showed that fibrin ECM contributes to hepatic inflammation during experimental ALD [37]. The mechanism(s) by which the ECM may regulate these earlier phases of ALD are currently not completely elucidated, but there are data supporting the hypothesis that altered matrix ECM may contribute to hepatic inflammation. For example, fibrin matrices have been shown to be chemotaxic and promote the activation of monocytes and leukocytes [81,82]. Furthermore, fibrin clots disrupt the flow of blood within the hepatic parenchyma (*i.e.*, hemostasis), the subsequent microregional hypoxia and hepatocellular death may directly (e.g., via HIF1α) and indirectly alter inflammatory cell signaling [83,84].

ECM not only serves as the physical structure, but also binds/interacts with several biomolecules that can directly or indirectly alter responses. One family of receptors that mediate these interactions is the integrins. Integrins transfer information from the ECM to the cell, allowing rapid and flexible responses to changes in the environment. Integrins play a myriad of roles within the body, including proliferation/angiogenesis, as well as inflammation and apoptosis [85,86]. These integrins are found on almost all cell-types in the several non-parenchymal cells in the liver. Therefore, altering the composition of the ECM has the potential to alter inflammatory signaling in liver via a myriad of mechanisms. Modulators of integrin function are in human clinical trials for non-hepatic diseases, and may ultimately be used in hepatic problems such as ALD; however, the ubiquity of expression of some integrins may make them difficult systemic pharmacologic targets.

## 3. Alcohol and Mechanisms of Lung Injury

### 3.1. Alcohol and Pulmonary Alcohol Metabolism

After its consumption, 95% of the alcohol ingested is absorbed by the body without changes to the molecule. The stomach absorbs approximately 20%, while the duodenum and small intestine absorb close to 75% of the consumed alcohol [87,88]. The remaining 5% is eliminated from the body, mainly through respiration, without any changes. Most of the absorbed alcohol is metabolized in the liver, but other tissues, such as the gastrointestinal mucosa, the brain, the spleen, and the lungs, also have the ability to metabolize alcohol. Alcohol that is not metabolized in the liver travels directly to the lungs by way of pulmonary circulation. The ingested alcohol itself can freely diffuse from the bronchial circulation and epithelium and vaporize into the conducting airways [88], as evident from the analysis of the breath test used to determine a person’s blood alcohol levels [89]. Interestingly, it is this vaporized alcohol which can cycle back into the airway lining fluid and affects the airway epithelium repeatedly. This creates a viscious “recycling” of high concentrations of alcohol and results in multi-exposures of the epithelium [88].

The above process allows for the lungs to be exposed to relatively large quantities of alcohol, especially in alcoholics. There, alcohol can be converted into fatty acids [90], and its products (e.g., acetaldehyde and CO_2_) be eliminated from the blood. The elimination rate of alcohol from the blood in a normal person is approximately 15 mg/100 mL/h [91]. As described above, direct effects of alcohol, or the products of its metabolism, include damage to the host respiratory defense mechanism, normal respiratory function, as well as other non-respiratory functions of the lungs, such as modification of circulating biologically active materials, filtration of blood and serving as a reservoir for the blood. Indirectly, alcohol and its metabolites may damage the endocrine system and disrupt the actions of hormones like cortisol, testosterone, growth hormone, and prolactin which, in turn, may affect pulmonary function or the function of related organs. For example, both alcohol and its metabolic products have been shown to interfere with glucose and lipid metabolism [90,92].

### 3.2. Dysregulated Pulmonary Inflammation

Inflammation is known as an adaptive response designed for restoring homeostasis and protection against infection and tissue injury [93,94]. In the normal setting, inflammation is thought of as a beneficial response and a protection against infection; however, if left uncontrolled, as seen in septic shock, inflammation can be harmful, as well as life threatening. As stated before, alcohol is known to affect many different inflammatory mediators. In lung, alveolar macrophages from alcoholic subjects secrete less TNFα when stimulated with lipopolysaccharide *in vitro* [95]. In animal models, chronic alcohol ingestion suppresses not only TNFα secretion, but also the release of other pro-inflammatory cytokines, reactive oxygen species, and reactive nitrogen species [96,97]. In parallel, alcohol induces the expression and activation of TGFβ, a potent pro-fibrotic and anti-inflammatory factor that may interfere with normal innate and adaptive immune responses which, in turn, render the alcoholic subject susceptible to both acute lung injury and serious pulmonary infections [98,99].

More recent experimental findings suggest that another fundamental defect in the “alcoholic lung” is impaired responsiveness to granulocyte/macrophage colony-stimulating factor (GM-CSF). Alveolar macrophages from alcohol-fed rats have decreased expression of GM-CSF receptors on their surface membrane and decreased expression and nuclear binding of the GM-CSF-dependent transcription factor PU.1 [27]. This is important because the GM-CSF priming of alveolar macrophages is absolutely required for innate immune functions including the release of TNFα and other pro-inflammatory cytokines, as well as for bacterial phagocytosis and regulation of Th1 and Th2 responses within the alveolar space [99]. Interestingly, treatment with recombinant GM-CSF via the upper airway restored GM-CSF receptor expression, PU.1 activation, and innate immune functions, including TNFα secretion, in alveolar macrophages of alcohol-fed rats [27], and prevented endotoxin-mediated acute lung injury [99,100]. Chronic alcohol ingestion is also linked to derangements in nutrition causing decreases in zinc levels in the alveolar space and within the alveolar macrophages [101]. In alcohol-fed rats there was a five-fold decrease in lung bacterial clearance compared to control-fed rats; however, dietary zinc supplementation normalized bacterial clearance, abrogated oxidative stress in the alveolar space, and increased nuclear binding of both PU.1 and Nrf2 in alveolar macrophages [102]. This is not surprising considering zinc’s many roles in enzymatic processes and other intracellular functions. Together, these studies suggest that alveolar macrophages are profoundly impacted by alcohol exposure. Some studies suggest that infiltrating *versus* resident macrophages respond differently to inflammatory stimuli [103,104], but how alcohol affects these processes in the lung is unknown. Neutrophil recruitment and function are also adversely affected by alcohol [105,106]. Impaired TNF-a production, decreased tissue migration through the down-regulation of cell adhesion molecules, and hyporesponsiveness to chemoattractants are some of the mechanisms implicated [19,106,107]. Although increased susceptibility to infection in the alcoholic lung is likely to be mostly due to impaired immune cell function, the fact that alcohol affects many non-immune cell populations (*i.e.*, epithelium, endothelium, fibroblasts) suggests that susceptibility to infection in the alcoholic lung is likely multifactorial.

The recognition that alcohol abuse dramatically increases the risk of acute lung injury has raised important questions regarding the underlying mechanisms involved in this process. Clinical and experimental studies provide overwhelming evidence that acute lung injury is mediated by an intense inflammatory response to an initial insult, such as sepsis or trauma [108]. The exuberant release of pro-inflammatory cytokines, including TNFα, IL-1β, IL-6, and IL-8, into the alveolar space stimulates a robust influx of activated neutrophils and other immune cells that appear to damage the otherwise “innocent bystander” lung [108]. If this paradigm is indeed true, then it begs the question as to how alcohol abuse, which by all accounts appears to suppress (not increase) the innate immune response in the lung, increases lung injury. As will be discussed later, we believe that alcohol promotes acute lung injury through the induction of oxidative stress, damage of the lung epithelium, and activation of tissue remodeling.

Like the liver, the alcoholic lung may also be more sensitive to damage/changes caused by inflammation. Chronic alcohol ingestion significantly alters the permeability of the alveolar epithelium [109]. This might be related to the overexpression of TGFβ. In the chronic alcohol-fed rat model, latent or inactive TGFβ protein expression was increased two-fold in lung tissue [110]. When compared with rats fed control diets, the chronic alcohol-fed rats released approximately five times more activated TGFβ into the airspace during endotoxemia. Further, bronchoalveolar lavage fluid from endotoxemic alcohol-fed rats was capable of inducing a significant permeability defect in intact alveolar epithelial monolayers derived from control-fed rats, and this permeability defect was completely inhibited by neutralizing antibodies to TGFβ1 [110]. Glutathione dietary supplementation also inhibited alcohol-induced TGFβ expression [111]. Taken together, these experimental findings indicate that chronic alcohol ingestion increases the expression of TGFβ1 in the lung which can directly increase epithelial permeability resulting in non-cardiogenic pulmonary edema, the hallmark of acute lung injury. Further support for the role of TGFβ in acute lung injury is evidence by the observation that animals lacking integrins involved in TGFβ activation are protected from experimental acute lung injury [112,113]. Chronic alcohol ingestion also alters the expression of cell-cell adhesion molecules important in maintaining the integrity of the epithelial barrier [105]. Claudin-1 and claudin-7, for example, are relatively specific to alveolar epithelial type I pneumocytes that form the vast majority of the alveolar epithelial barrier *in vivo*, and increases in claudin-5 have been associated with increased epithelial permeability in other systems. Therefore, changes in claudin expression in the alveolar epithelium may produce a “leakier” phenotype that renders the alcoholic lung susceptible to alveolar flooding during acute inflammatory stresses [109].

### 3.3. Oxidative Stress

As stated before, oxidative stress occurs when the normal balance between pro-oxidants and anti-oxidants is shifted such that the increase of oxidants leads to damage or destruction of tissue and tissue function [114]. In the chronic alcoholic lung, oxidative stress is a key factor in the increased risk of ARDS [30,115,116,117]. In animals exposed to chronic alcohol (6–8 weeks), lung glutathione levels decrease dramatically thereby depleting the lung of important anti-oxidant capacity [30]. In addition, studies show that chronic alcohol exposure results in alveolar macrophage dysfunction due to glutathione depletion in the lung [30,99,118,119,120]. Specifically, the ability of alveolar macrophages to bind and internalize inactive *Staphylococcus aureus* is impaired, but treatment with a glutathione precursor improves macrophage phagocytosis *in vivo* [119]. Similar depletion of lung glutathione levels have been documented in the bronchoalveolar lining fluid of alcoholic subjects [34] and in their alveolar macrophages [118].

More revealing is the observation that anti-oxidant treatments like *N*-acetylcysteine and procysteine are protective in the experimental animal [30]. However, anti-oxidants have failed to affect outcomes significantly in the setting of acute lung injury in humans suggesting a more complex scenario. One potential mechanism for alcohol-induced oxidative stress is up-regulation of the Nox family of proteins that comprise multicomponent, membrane-associated Nox enzymes that generate reactive oxygen species [121]. Previous studies showed that, in whole lung tissue, chronic alcohol ingestion increases the expression of Nox2, the classical phagocytic oxidase essential for reactive oxygen species generation during respiratory burst [122]. Perhaps strategies designed to reduce alcohol-mediated increases in Nox2 expression and activity may provide a more effective therapeutic approach for attenuating alcohol-induced oxidative stress and dysfunction [123]. However, as mentioned above (see Section 2.2 and Section 3.2), alcohol exposure causes both impaired immune surveillance and enhanced inflammatory organ damage; as such, targeting NOX should be employed with care as NOX deficiencies impair immune surveillance against infection [124].

It is well known that oxidant stress can be generated through a number of mechanisms ranging from aberrant function of NADPH oxidase, alterations in glutathione transport, and the generation of reactive oxygen and nitrogen species, among other mechanisms [125]. Reduction of reactive oxygen species is driven by NADPH-dependent thioredoxin reductase and glutathione disulfide reductase. Others have tested the role of these mechanisms in the expression of matrix genes and the activation of pro-fibrotic growth factors like TGFβ1 [126]. Another mechanism of oxidative stress is related to the oxidation of thiol disulfide pairs such as cysteine and cystine (Cys/CySS), glutathione, and glutathione disulfide (GSH/GSSG), and thioredoxin, which results in alterations in their redox potential, hereafter termed “Eh.” An intriguing aspect of the Eh Cys/CySS is that it is operative extracellularly, while the Eh GSH/GSSG and thioredoxin predominate intracellularly (with thioredoxin predominating in the mitochondria). The Cys/CySS thiol disulfide redox couple is the predominant low molecular weight thiol disulfide pool found in plasma, and it controls intracellular levels of glutathione [127]. Interestingly, Jones [128] and others have documented oxidation of the Eh Cys/CySS in alcohol abuse and they have suggested that these changes in the redox potential of extracellular thiol pairs can serve as independent transducer of oxidant stress. The novelty of this signaling mechanism is that it can trigger intracellular redox signaling by oxidation of membrane-bound proteins, and this oxidation can trigger the down-stream generation of reactive oxygen species.

Exactly how Eh Cys/CySS affects cell function is unknown. Jones and his colleagues showed activation of the p44/p42 MAPK pathway by extracellular Eh Cys/CySS in Caco-2 cells, and this was associated with increased cell proliferation [129]. In endothelial cells, an oxidized Eh Cys/CySS was sufficient to activate cellular reactive oxygen species generation. More recently, and relevant to the lung, Ramirez *et al.* demonstrated that an oxidized extracellular Eh Cys/CySS results in activation of TGFβ1/Smad3 signaling in lung fibroblasts, and that this was associated with induction of fibronectin production, the expression of smooth muscle cell markers (*i.e.*, α-smooth muscle actin), and increased proliferation [130]. Others have also begun to evaluate the redox potential found in animals exposed to bleomycin, a well-known model for lung injury. These studies reveal that bleomycin-induced lung injury further enhances oxidation of the Cys/CySS and GSH/GSSG redox potentials [131]. In other work, we showed that Eh Cys/CySS controls pro-inflammatory cytokine levels [132]. These are interesting observations considering that Cys availability and extracellular Eh Cys/CySS are dependent on nutrition, disease, and environmental exposures. Thus, oxidation of the redox potential of distinct thiol disulfide couples is likely to represent yet another pathway by which alcohol could exert its detrimental effects.

### 3.4. Altered ECM Metabolism

The ECM of the lung is composed of a meshwork of fibers that provide essential functions such as structural stability, barrier partitioning, storage of growth factors (e.g., TGFβ1), and regulation of many critical cellular functions, including cell signaling, proliferation, apoptosis, wound healing and fibrosis. Proteolytic fragments of the ECM themselves have discrete biological functions, the most-studied example being the chemotactic activity imparted by elastin, collagen, fibronectin, and laminin fragments.

Since alcohol-related liver disease is known to be associated with dramatic alterations in connective tissue deposition and scar formation (cirrhosis), Roman and others investigated the effects of alcohol on connective tissue expression, deposition, and degradation in lung. In rats fed with alcohol chronically (6–8 weeks), they demonstrated activation of lung tissue remodeling as highlighted by the increased expression of pro-fibrotic factors such as TGFβ1 [110], which we have already highlighted as an important driver of acute lung injury. Increased expression and activity of matrix-degrading proteases such as the gelatinases MMP-2 and MMP-9 were also documented and these were associated with increased fragmentation of collagen type IV in bronchoalveolar lavage fluid [29]. Alcohol also stimulates lung fibroblasts to express fibronectin, a matrix glycoprotein implicated in lung injury and repair [28]. This glycoprotein is highly expressed in embryonic tissues, but in the adult, its expression is limited to the liver and large vascular structures. However, in injured tissues, fibronectin expression increases. In this setting, fibronectin is thought to provide a temporary scaffold for the re-epithelialization of denuded basement membranes [133], promote fibroproliferation [134], and serve as a non-immune opsonin to clear infections, while its fragments are chemotactic to inflammatory cells [135]. Also noticeable is the observation that alcohol increases STAT5 phosphorylation in response to IL-13, which has been implicated in tissue fibrosis [26].

The above work prompted the search for evidence of activation of lung tissue remodeling in humans chronically exposed to alcohol. This work has been hampered by the fact that, other than in the setting of pulmonary infection and/or acute lung injury, alcoholics do not show clinical signs of pulmonary dysfunction at baseline, thereby limiting the need for invasive procedures that could facilitate the collection of clinical samples for study. However, one study reported increased fibronectin and MMP mRNA expression in the alveolar macrophages of alcoholics [136,137]. Furthermore, the lavage fluid of alcoholics was capable of stimulating fibronectin expression in fibroblasts when compared to control. What is intriguing about these studies is that the subjects recruited showed no evidence of lung disease.

It is important to emphasize that chronic alcohol exposure leads to activation of tissue remodeling without significant effects on overall lung architecture. In other words, in spite of the many cellular abnormalities detected and the alterations noted in expression of tissue remodeling genes in both animal models and humans, the overall architecture of the lung seems unaltered. In fact, detection of activation of tissue remodeling in the setting of chronic alcohol exposure is only possible by immunohistochemistry or other more sophisticated tests. Thus, chronic alcohol exposure leads to what we have termed “transitional remodeling”, a process characterized by alterations in the expression of distinct tissue remodeling genes (e.g., fibronectin) without causing overt changes in lung architecture and pulmonary mechanics. This concept is consistent with the observation that alcoholics do not manifest “lung disease” unless affected by lung infection or acute lung injury. This concept may be re-stated as a “first hit-second hit hypothesis” with alcohol (first hit) promoting transitional remodeling and other abnormalities that do not lead to overt lung dysfunction without a second hit such as sepsis or aspiration of gastric contents.

These studies also raise two important questions—what is the role of transitional remodeling in lung and is it involved in alcohol-related increased susceptibility to acute lung injury? Although these questions remain unanswered, a study performed by Brown *et al.* revealed that alveolar type II cells harvested from alcohol-treated animals were capable of depositing a fibronectin-rich matrix *ex vivo*. Monocytic cells cultured atop of these newly derived matrices expressed more interleukin-1β than cells cultured on matrices derived from control cells [137]. These effects were inhibited by antibodies against the fibronectin integrin receptor α5β1 further emphasizing the role of fibronectin. Consistent with a role for oxidant stress, the investigators also demonstrated that cells harvested from animals concomitantly treated with the anti-oxidants *N*-acetylcysteine and procysteine did not produce a fibronectin-rich matrix capable of inducing IL-1β expression. In another report, Koval and colleagues reported that fibronectin-rich matrices alter the relative expression of cell-cell adhesion molecules of the claudin family leading to alterations in epithelial cell permeability *in vitro* [138]. Together, these and other studies suggest that even minor changes in the relative composition of the lung matrix can affect the function of resident and incoming cells after lung injury thereby promoting an exaggerated response that can lead to acute lung injury after an insult.

## 4. Gut:Liver:Lung Axis in Alcohol-Induced Organ Pathology

### 4.1. Multiorgan Dependencies in Disease

It is now clear that human diseases are generally a multi-stage, multi-hit process; it is therefore not surprising that multiple cells within a target organ contribute to disease pathology. However, although this assumption is almost universally accepted, the contribution of signals outside the target organ to disease pathology has been slower to be accepted in several cases. The clarity of hindsight may make this difficult to understand, but should be taken into context of the research at the time. In the advent of the modern molecular biology era, more emphasis was placed on distinct signaling cascades within the cell; as such, more and more research was performed in purified cultured cells. Although it is understandable that this approach is useful for limiting the “black box” of the experimental design, several aspects of the *in vivo* situation simply cannot be recapitulated in this paradigm. The development of more specific molecular techniques that can be employed *in vivo* or *in situ* (e.g., transgenic mice, viral gene delivery and “knockdown” approaches) have enabled researchers to again test specific hypotheses in intact organs/animals. The further advance of temporal and/or locational control of gene expression (e.g., with conditional transgenics) has further enabled research to be performed on a system level. These advances coevolved with the era of ‘omics research in which large amounts of data can be simultaneously analyzed for trends and effects. The net result is that system level analyses of disease, and organ-organ interactions are again gaining attention of the research community.

### 4.2. Alcohol and the Gut:Liver Axis: Historical Perspectives

Interaction between the gut and liver is long established and has been recognized for more than a hundred years. As early as 1893, Pavlov observed that low levels of intestinally-derived “toxins” are normally present in the portal blood and are cleared by the liver [139]. Indeed, these experiments led Pavlov to propose that gut-derived toxins could be responsible for systemic disease [139]. More than half a century later, several studies suggested that experimental fibrosis caused by choline deficiency was dependent on intestinal bacteria. For example, Rutenburg and colleagues showed that non-absorbable antibiotics protected against diet-induced cirrhosis in rats [140], however, the protection conferred by the non-absorbable antibiotic neomycin was lost when purified LPS was administered concomitant with neomycin [141]. These experiments demonstrated that cirrhosis in this model was due to endotoxin rather than bacterial activity itself. The causal role of endotoxin in liver injury was not confined to models of diet-induced cirrhosis, but was further established in studies using acute hepatotoxins such as CCl_4_ [142,143]. The development of the Limulus lysate assay allowed for the detection of LPS in human plasma in the context of liver disease [144]. Such studies revealed high systemic levels of LPS in both acute and chronic liver diseases [145,146].

Early studies that tested the concept of the gut/liver axis demonstrated that alcohol consumption was a contributing factor to endotoxemia, with systemic endotoxemia occurring significantly more often in alcoholic cirrhotics compared to their non-alcoholic counterparts [147]. These researchers demonstrated that acute alcohol transiently increased systemic levels of endotoxin. Studies using experimental models confirmed the detrimental effect of gut-derived endotoxin in rats by protecting against of alcohol-induced liver injury with antibiotics [148]. Almost 25 years later, the advent of genetically-modified mice (e.g., knockout mice) rapidly increased our mechanistic insight into the role of LPS in alcohol-induced liver injury and validated the original idea [149,150,151]. The gut-liver axis and enteric dysbiosis is now a generally-accepted component in the development of ALD [152,153], as well as other liver diseases [154].

### 4.3. Alcohol and the Gut:Lung Axis: Historical Perspectives

As mentioned before, chronic alcohol abuse is associated with increased incidence of acute lung injury [23]. Interestingly, mortality in acute lung injury in the setting of hepatic failure is almost 100% [155,156]. In experimental models of liver injury, vascular permeability and leukocyte activation in lungs are increased [157]. These, and other, studies have led some investigators to postulate that liver-lung interactions represent an important aspect of the pathophysiology of these disorders, particularly in sepsis. In a study performed in an *in situ* perfused piglet preparation, Brigham and collaborators tested this idea [158]. This model allows for the separation of the lung and liver circulations. The investigators showed that endotoxemia increased pulmonary vascular resistance in the lung circuit as well as when both liver and lung were perfused. However, significant hypoxemia and pulmonary edema were only observed when both lung and liver were perfused. The latter was associated with increases in the transcription factor nuclear factor kappa beta (NF-kB), the cytokines IL-6 and TNFα, and a marker of oxidant stress, 8-isoprostane. The investigators concluded that the direct effects of endotoxemia in lung include vasoconstriction and leukocyte sequestration, but not lung injury; lung injury is only elicited when the lung interacts with the liver leading to a severe inflammatory response and oxidative injury. Further studies in this model revealed that endotoxemia causes mitochondrial dysfunction as has been reported for liver and other organs [159]. Importantly, the investigators found that procysteine was protective in this model while *N*-acetylcysteine was less effective. Further work revealed that endotoxin resulted in oxidation of the mitochondria-specific protein thioredoxin-2, while procysteine reduced thioredoxin-2 oxidation. The above observations not only implicate oxidant stress in alcohol-related liver and lung dysfunction, but point to redox signaling in specific intracellular compartments as a key target for these processes.

### 4.4. Alcohol and Gut:Liver:Lung Axis: New Areas to Explore

**Figure 1:**
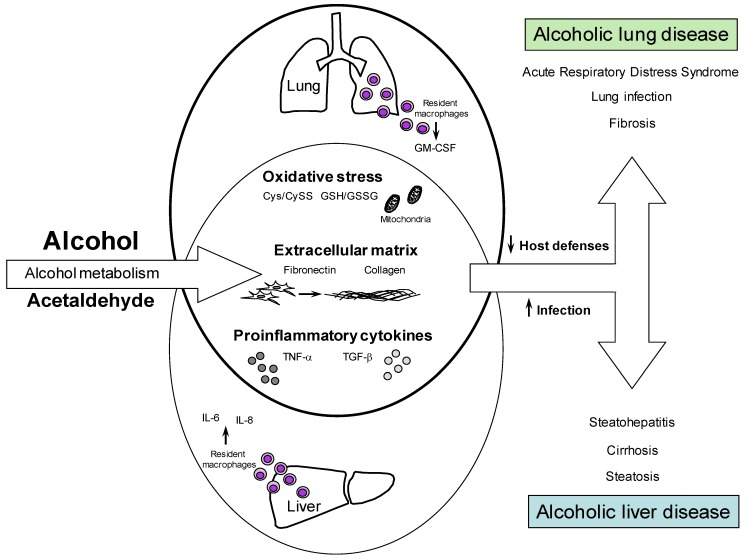
Effect of alcohol on the gut/liver/lung axis. Alcohol induces tissue damage in liver and lung through shared mechanisms of action that promote the progression of alcoholic liver and lung disease. Resident macrophages found in both the liver and lung play key roles in mediating inflammatory responses including the induction of cytokines such as IL6, IL-8, TNF-α, TGF-β, and GM-CSF. Activation of tissue remodeling, with enhanced deposition of extracellular matrix components like fibronectin and collagen, and increased oxidative stress also contribute to tissue injury and organ dysfunction. Ultimately, alcohol-induced damage to both organs results in the impairment of host defense mechanisms and susceptibility to infection, tissue injury and disease.

Although less well-characterized, several studies indicate interdependence between liver and lung, potentially via mediators released from the gut (see Figure 1). For example, mortality in ARDS patients with hepatic failure is almost 100% [156]. Furthermore, pulmonary injury induced by systemic endotoxin can be altered by mediators released from the liver (e.g., TNFα) [157,160]. Recently, depletion of systemic TNFα (etanercept) was demonstrated to prevent pulmonary injury in a mouse model of alcohol-enhanced ALI [161]. Furthermore, in an elegant study, Siore *et al.* [158] demonstrated that LPS-induced lung damage requires perfusion with the liver. The most likely source of systemic LPS (absent sepsis) is the GI tract, which indirectly potentially links gut damage with lung damage; however, the role of liver in this process is poorly understood.

## 5. Summary and Conclusions

Much is understood about the mechanisms by which alcohol damages the liver and the lung. However, this insight has yet to translate to an FDA-approved therapy to blunt, prevent, or reverse the progression of disease. As summarized in Section 2 and Section 3 above, although the liver and lung share many similar responses to alcohol, whether these are parallel or interdependent mechanisms remains to be determined experimentally. Furthermore, mechanisms that have been identified to contribute to one organ in alcohol pathology should be tested in the other. As mentioned above (see Section 4.1), the advent of molecular approaches in which the expression of gene can be altered in a target organ (e.g., conditional knockouts) allow us to identify new mechanisms and to address the interplay between organs in target pathology more effectively. This new area of research should be pursued with the concept that alcohol-induced pathology is a systemic disease. Importantly, should shared, interdependent mechanisms be identified, these findings could translate to new therapies that may be more effective for treating alcohol-induced tissue pathology in both liver and lung.
